# Emotional and Situational Acceptance Across the Lifespan: A Novel Scale

**DOI:** 10.1093/geroni/igaa057.1477

**Published:** 2020-12-16

**Authors:** Hannah Wolfe, Derek Isaacowitz

**Affiliations:** Northeastern University, Boston, Massachusetts, United States

## Abstract

Self-reported emotional well-being tends to increase with age (Charles & Carstensen, 2007), but evidence for age differences in emotion regulation strategies is mixed (Livingstone & Isaacowitz, 2019), and the strategy of acceptance, in particular, is relatively understudied. Acceptance involves the deliberate decision to not alter a situation or one’s emotional response to it, and older adults report greater use of general acceptance (Shallcross, Ford, Floerke, & Mauss, 2013). Yet, no current scale distinguishes between situational and emotional acceptance; general acceptance is typically measured using a subscale of the Kentucky Inventory of Mindfulness Skills (KIMS; Baer, Smith, & Allen, 2004), which assesses judgments of emotions and thoughts. Therefore, a 6-item measure of situational acceptance was developed and administered to 24 younger adults (age 18-25) and 30 older adults (age 55+) on Amazon Mechanical Turk, along with the KIMS accepting subscale and Mindful Attention Awareness Scale (MAAS; Brown & Ryan, 2003). The situational acceptance scale achieved good reliability (α=.721) and significantly correlated with the MAAS (r= .301, p=.027) and KIMS (r= .466, p<.001). Older adults tended to rate themselves as significantly higher on situational acceptance (M=29.83, SD=5.17) than younger adults (M=25.13, SD=5.72; t=-3.171, p=.003), and this pattern held for the MAAS and KIMS. These results confirm prior work suggesting older adults engage in acceptance more often than younger adults and expand this finding to situational, not just emotional, acceptance. Furthermore, skills related to mindfulness and acceptance appear to greatly overlap and may increase over the lifespan.

